# The Medical Library Association

**Published:** 1903-01

**Authors:** 


### THE MEDICAL LIBRARY ASSOCIATION.

A MEDICAL library association has been organized as an institution separable from the Atlanta Society of Medicine, from which it sprung. The officers are: Dr. Michael Hoke, President; Dr. ————, Vice-President; Secretary and Treasurer, Dr. E. Bates Block; Executive Committee, Drs. V. O. Hardon, W. S. Elkin and Alex. W. Stirling. The annual dues are $5, and any one practicing medicine or any of the allied sciences is eligible to membership. The repository of books will be the Carnegie Library, where ample space has been secured.

---

THE officers of the Atlanta Society of Medicine elected for the ensuing year are: President, Dr. L. P. Stephens; Vice-President, Dr. Michael Hoke; Secretary, Dr. W. B. Emery; Treasurer, Dr. E. Van Goidtsnoven; Member of the Executive Committee, Dr. W. S. Goldsmith.

